# Effect of Human Burn Wound Exudate on *Pseudomonas aeruginosa* Virulence

**DOI:** 10.1128/mSphere.00111-15

**Published:** 2016-04-27

**Authors:** Manuel R. Gonzalez, Betty Fleuchot, Leonardo Lauciello, Paris Jafari, Lee Ann Applegate, Wassim Raffoul, Yok-Ai Que, Karl Perron

**Affiliations:** a Microbiology Unit, Department of Botany and Plant Biology, Sciences III, University of Geneva, Geneva, Switzerland; b Plastic, Reconstructive & Hand Surgery, Unit of Regenerative Therapy, Lausanne University Hospital, Lausanne, Switzerland; c Service of Adult Intensive Care Medicine & Burns, Lausanne University Hospital, Lausanne, Switzerland; Swiss Federal Institute of Technology Lausanne

**Keywords:** burn wound exudate, *Pseudomonas aeruginosa*, virulence factors, growth

## Abstract

Burn wound sepsis is currently the main cause of morbidity and mortality after burn trauma. Infections by notorious pathogens such as *Pseudomonas aeruginosa*, *Staphylococcus aureus*, and *Acinetobacter baumannii* impair patient recovery and can even lead to fatality. In this study, we investigated the effect of burn wound exudates (BWEs) on the virulence of those pathogens. BWEs were collected within 7 days after burn trauma from 5 burn patients. We first monitored their effect on pathogen growth. In contrast to *A. baumannii* and *S. aureus*, *P. aeruginosa* was the only pathogen able to grow within these human fluids. Expression of typical virulence factors such as pyocyanin and pyoverdine was even enhanced compared the levels seen with standard laboratory medium. A detailed chemical composition analysis of BWE was performed, which enabled us to determine the major components of BWE and underline the metabolic modifications induced by burn trauma. These data are essential for the development of an artificial medium mimicking the burn wound environment and the establishment of an *in vitro* system to analyze the initial steps of burn wound infections.

**IMPORTANCE** Microbial infection of severe burn wounds is currently a major medical challenge. Of the infections by bacteria able to colonize such injuries, those by *Pseudomonas aeruginosa* are among the most severe, causing major delays in burn patient recovery or leading to fatal issues. In this study, we investigated the growth properties of several burn wound pathogens in biological fluids secreted from human burn wounds. We found that *P. aeruginosa* strains were able to proliferate but not those of the other pathogens tested. In addition, burn wound exudates (BWEs) stimulate the expression of virulence factors in *P. aeruginosa*. The chemical composition analysis of BWEs enabled us to determine the major components of these fluids. These data are essential for the development of an artificial medium mimicking the burn wound environment and for *in vitro* analysis of the initial step in the development of burn wound infections.

## Abstract (precis)

Microbial infection of severe burn wounds is currently a major medical challenge. Of the infections by bacteria able to colonize such injuries, those by *Pseudomonas aeruginosa* are among the most severe, causing major delays in burn patient recovery or leading to fatal issues. In this study, we investigated the growth properties of several burn wound pathogens in biological fluids secreted from human burn wounds. We found that *P. aeruginosa* strains were able to proliferate but not those of the other pathogens tested. In addition, burn wound exudates (BWEs) stimulate the expression of virulence factors in *P. aeruginosa*. The chemical composition analysis of BWEs enabled us to determine the major components of these fluids. These data are essential for the development of an artificial medium mimicking the burn wound environment and for *in vitro* analysis of the initial step in the development of burn wound infections.

## INTRODUCTION

*Pseudomonas aeruginosa* is a ubiquitous gammaproteobacterium found in different environmental niches such as soil and water. As an opportunistic pathogen, it also causes severe infections in mammals and other animals and in plants (1). The pathogenicity of *P. aeruginosa* is mediated by its capacity to produce a large range of virulence factors and is strengthened by its intrinsic resistance to environmental stresses and xenobiotic agents such as antibiotics, disinfectants, and heavy metals (2). Taking the data together, it has been shown that these factors allow the pathogen to establish efficient invasion, colonization, and persistence inside the host organism (3, 4).

The gene expression profile of *P. aeruginosa* during the infection process is tightly regulated and requires the activation of cell density-dependent mechanisms called quorum-sensing (QS) mechanisms (5, 6). The induction of such systems involves the production of signaling molecules, the autoinducers, and regulatory proteins. The detection of the autoinducer by its cognate transcriptional regulator induces a positive-feedback loop leading to the autoinduction of the QS system and to a coordinated change of the bacterial population physiology. These regulatory systems are involved in virulence factor production and biofilm formation and in the switching of the bacterium to its pathogenic state (7). Among the major virulence factors produced, the blue pigment pyocyanin triggers proinflammatory activity (8) and the QS-independent regulated pigment pyoverdine is a siderophore involved in iron chelation and acquisition (9). There is also the secreted protease elastase, which mainly contributes to the destruction of elastin, a component of the host tissues (10). The major complications due to *P. aeruginosa* infections are observed in organisms with compromised natural defenses. These situations are found in, for example, immunocompromised or burn patients after the introduction of foreign materials, such as catheters, into the body (2, 11) or when a physiological function is altered, as in the case of mucus accumulation in lungs of cystic fibrosis (CF) patients (7). Indeed, following an acute phase, *P. aeruginosa* often establishes a chronic infection via the formation of a biofilm which prevents or delays healing of the patient and which can lead to fatality (12, 13).

Severe burn injuries are part of the most devastating form of trauma, including loss of the skin barrier and tissue destruction, and require immediate and specialized medical care to maintain homeostasis (13, 14). In addition to body temperature maintenance, the prevention of fluid loss through supplementation of liquid and electrolytes represents critical parameters for positive vital prognosis (15, 16). Indeed, tissue damage at burn wound sites causes the loss of the biological fluids defined as burn wound exudates (BWEs) (reviewed in reference 17). While the wound bed is accessible, biological dressings based on collagen matrices are applied on the wound. These bandages were optimized by the incorporation of progenitor cells whose growth factor secretion promotes wound healing (18). BWEs are fluids enriched in proteolytic elements (19) and contain several immune molecules (20). Through their production and composition, they greatly influence the overall state of the patient and the wound healing process in particular (21). Despite the need for study of crucial biological functions, no artificial formulation is currently available that mimics BWE, contrasting with other well-studied biological fluids such as CF sputum (22).

Burn wounds are complex microenvironments where infections by bacterial pathogens such as *P. aeruginosa* or *Staphylococcus aureus* represent major concerns in patient treatment (11, 13). The understanding and characterization of the bacterial physiology in relation to burn wound exudate composition are of high interest for the development of novel strategies to prevent and cure bacterial infections. In this study, we focused on the analysis of the pathogenic traits of *P. aeruginosa* PAO1 in BWE and linked these findings to an understanding of the physicochemical and biological properties of those exudates to eventually propose an artificial burn wound exudate medium for the establishment of an *in vitro* system to analyze the initial steps of burn wound infections.

## RESULTS

### Burn wound exudate collection and formulation of an exudate mix.

Five major burn patients (with superficial or deep second-degree burns) admitted to the Lausanne University Hospital Burn Intensive Care Unit (BICU), between February and October 2014, were included in the present study (for more details, see Table 1). Burn wound exudates (BWEs) were collected twice a day (morning and evening) by vacuum aspiration through a closed and sterile collection system (method submitted for publication), aliquoted, and stored at −80°C until analysis. The volumes of BWE produced were varying over time until skin grafting took place. In order to avoid a bias caused by antimicrobial treatments, a selection of the BWEs free of antibiotics was carried out. Only seven BWEs fit within this criterion. All presented a color variation from light yellow to orange, and an alkaline pH was reported for each sample (Fig. S1A), as already described in the literature (23). To deal with the small volumes available for each BWE selected, a reference exudate mix (referred to here as “exudate mix”) was established based on a mixture of the 7 BWEs in equal volumes.

10.1128/mSphere.00111-15.1 Figure S1  Visual aspect and pH values of BWEs. Data represent the visual aspect and pH values of the 7 BWEs used to elaborate the reference mix of exudates (A) composed of all the nonantibiotics containing BWE and the 15 BWEs used for the physicochemical analysis (B). Exudates were collected in the morning (M) and evening (E) at different days after the burn injury. Samples 7 Ml (morning, left) and 7 MR (morning, right) were collected in the morning of day 7 postinjury from the left and right leg, respectively. Download Figure S1, TIF file, 0.4 MB. Copyright © 2016 Gonzalez et al. 2016 Gonzalez et al. This content is distributed under the terms of the Creative Commons Attribution 4.0 International license.

**TABLE 1:** Clinical characteristics of the burn patients^a^

Patient	Sex	Age (yrs)	Burn characteristic		Sampling period (no. of days)
			Cause	TBSA (%)	
Pt 3	M	37	Flame	60	8
Pt 4	M	85	Flame	42	3
Pt 6	M	19	Flame	47	8
Pt 7	M	49	Flame	15	3
Pt 11	M	55	Flame	23	4

a After admission of the burn patients (Pt) at the Lausanne Burn Center, sex (M, male) and age were recorded. The total body surface area (TBSA) and severity of the burn were evaluated at the intensive care unit. Burn wound exudates were collected using a negative-pressure dressing system for several days as indicated in Materials and Methods.

### Gram-positive and gram-negative bacterium growth in exudate mix.

**FIG 1:**
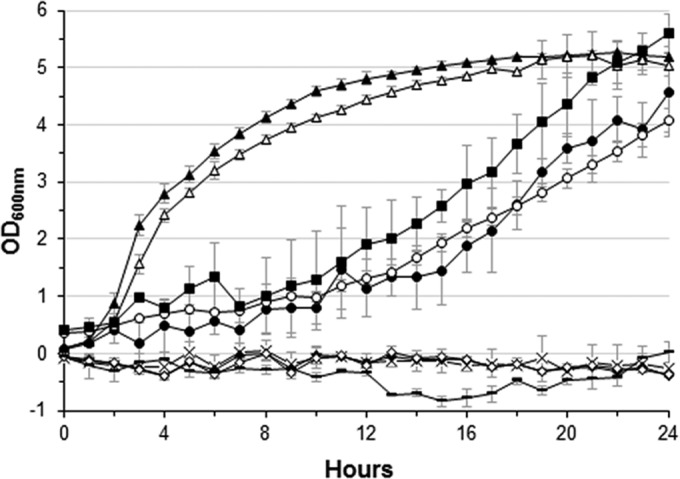
Growth of burn wound pathogens in burn wound exudate. Growth of *E. coli* MG1655 X, (×), *S. aureus* ATCC 29213 (◆) and USA300 (◊), *A. baumannii* ATCC 19606 (**─**), and *P. aeruginosa* PAO1 (●), PA14 (ο), and PA25688 (▪) was monitored over 24 h in burn wound exudate mix. *P. aeruginosa* PAO1 growth was also monitored in LB media at pH 7.0 (▴) and pH 9.0 (▵) as a control.

Wound infections can be caused by polymicrobial pathogen proliferation (24). To investigate whether common burn wound pathogens could develop within BWEs, we first monitored growth of *Pseudomonas aeruginosa* PAO1, *Staphylococcus aureus* USA300, and *Acinetobacter baumannii* ATCC 19606 in exudate mix and compared their growth curves to those seen under Luria-Bertani (LB) control conditions at pH 7.0 (standard LB pH) and pH 9.0 (exudate mix pH). Surprisingly, in contrast to *P. aeruginosa* PAO1, *S. aureus* USA300 and *A. baumannii* ATCC 19606 were not able to grow in the exudate mix (Fig. 1; see also Fig. S2A in the supplemental material). However, the *P. aeruginosa* PAO1 growth rate was lower (doubling time of 4.3 h) in the exudate mix than under the control LB pH 7.0 and LB pH 9.0 conditions, where doubling times of 44.2 min and 55.5 min, respectively, were seen. Nevertheless, the optical density (OD) values after 24 h appeared to be similar (Fig. 1). In order to determine whether BWE could function as a selective medium for some bacterial strains, we monitored the growth of other Gram-positive and Gram-negative bacterial strains: *S. aureus* ATCC 29213, *Escherichia coli* MG1655, and *P. aeruginosa* strains (PA14 and a clinical isolated strain, PA25688). Interestingly, none of the *E. coli* and *S. aureus* strains were able to growth in exudate mix (Fig. 1) whereas their growth rates were not affected under the LB medium conditions (see Fig. S2A in the supplemental material). In contrast, all *P. aeruginosa* strains exhibited similar positive growth kinetics, showing that the human burn exudates provide all the nutritional cues requisite for *P. aeruginosa* development, which might explain why this organism is a predominant pathogen in burn victims.

10.1128/mSphere.00111-15.2 Figure S2  Counting and growth of the burn wound pathogens. (A) Bacterial growth curves, in LB media (pH 7.0 [left panel] and pH 9.0 [right panel]), are represented as follows: *P. aeruginosa* strains PAO1 (▲), PA14 (○), PA25688 (■), *E. coli* MG1655 X, (×), *S. aureus* ATCC 29213 (◆), *S. aureus* USA300 (◇), and *A. baumannii* ATCC 19606 (─). (B) Counts of bacterial strains (*P. aeruginosa* PAO1 [lanes 1], *E. coli* MG1655 [lanes 2], *S. aureus* ATCC 29213 [lanes 3], *S. aureus* USA300 [lanes 4], and *A. baumannii* ATCC 19606 [lanes 5]) were determined under the following conditions. Bacteria were cultured in LB media or exudate mix over 24 h, and serial dilutions were plated on LB agar at h 0 (t0) and h 24 (T24). (C) Quantification of fold change in bacterial populations after 24 h of growth in exudate mix. Download Figure S2, TIF file, 0.8 MB. Copyright © 2016 Gonzalez et al. 2016 Gonzalez et al. This content is distributed under the terms of the Creative Commons Attribution 4.0 International license.

To further explore whether the absence of *A. baumannii*, *E. coli*, and *S. aureus* growth was linked to a bacteriostatic or a bactericidal activity of the BWE, the amount of viable cells was determined at time zero (t0) and after 24 h of growth (T24) in exudate mix (see Fig. S2B in the supplemental material). Although the CFU counts were similar for *E. coli* MG1655 and *A. baumannii* ATCC 19606 at t0 and T24, no CFU could be observed for either of the *S. aureus* strains at 24 h (see Fig. S2B and C). Taken together, these results demonstrated that the BWE produced by the human body is capable of inhibiting, *in vitro*, the growth of some pathogenic and nonpathogenic bacterial species such as *S. aureus*, *A. baumannii*, and *E. coli* but not *P. aeruginosa*. Moreover, the inhibition pattern could reach even a bactericidal effect for the *S. aureus* bacterial cells.

Finally, in order to characterize the *P. aeruginosa* bacteria that are able to grow in BWE, cells (at 24 h) were reinoculated in a fresh exudate mix. No difference in growth kinetics and doubling times was observed (see Fig. S3 in the supplemental material), suggesting that the dividing bacteria observed in BWE do not correspond to the selection of mutants able to growth in this particular medium. All things considered, we decided to further investigate the virulence factor production and the physiology of *P. aeruginosa* PAO1 in BWE.

10.1128/mSphere.00111-15.3 Figure S3  *P. aeruginosa* PAO1 reinoculation in burn wound exudate. Growth of *P. aeruginosa* PAO1 (●) was monitored over 24 h in burn wound exudate mix followed by reinoculation in fresh exudate mix and further incubation for 24 h (○). *P. aeruginosa* PAO1 growth was also monitored in LB media (pH 7.0 [▲] and pH 9.0 [△]) as a control. Download Figure S3, TIF file, 0.1 MB. Copyright © 2016 Gonzalez et al. 2016 Gonzalez et al. This content is distributed under the terms of the Creative Commons Attribution 4.0 International license.

### Exploration of *P. aeruginosa* PAO1 physiology in burn wound exudates. (i) Biofilm formation.

**FIG 2:**
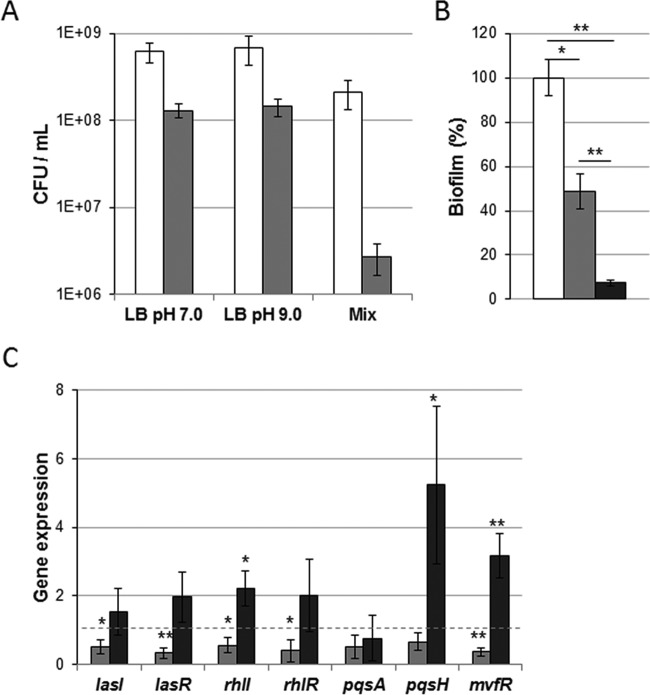
*P. aeruginosa* PAO1 biofilm production and QS gene expression in burn wound exudate. (A) CFU levels of planktonic (white bars) and adherent (gray bars) cells were quantified (in milliliters) after 24 h of growth under static conditions. Data represent averages of results of three experiments, and standard deviations as well as *P* values are indicated. (B) Biofilm formation by PAO1 was measured after 24 h of static growth in LB at pH 7.0 (white bar), LB at pH 9.0 (gray bar), and exudate mix (black bar). Values were normalized to OD_600_, and statistics were calculated from the results of three independent experiments performed in triplicate. (C) Expression levels of the quorum-sensing systems *lasI* and *lasR*, *rhlI* and *rhlR*, *pqsA*, and *pqsH* and *mvfR* were analyzed by qRT-PCR performed on material extracted from PAO1 cultures at an OD_600_ of 2.0 and were normalized to conditions of LB at pH 7.0 (dashed line). Statistics are indicated using *P* values of <0.05 (*) and *P* <0.01 (**).

*P. aeruginosa* pathogenesis depends on its capacity to form biofilms (reviewed in references 25 and 26). These complex structures play a crucial role in the adherence and maintenance of the pathogen on host surfaces. Biofilms also contribute to the overall resistance to antibiotics and immune factors and have been shown to increase the persistence of the bacterial community (25, 26). The fraction of adherent bacteria was quantified and compared to the fraction of planktonic bacteria. Results showed a level of adherent bacteria that was 77 times lower during growth in exudate mix (Fig. 2A), whereas the levels were only 5 times lower under the LB pH 7.0 and LB pH 9.0 conditions. The lower bacterial adherence observed in BWE was confirmed through the quantification of biofilm formed by *P. aeruginosa* PAO1 (Fig. 2B). The level of biofilm produced by *P. aeruginosa* in the exudate mix was 10% of the level produced under the LB pH 7.0 conditions. Although the ratios of biofilm to bacteria were similar under the BWE and the LB pH 7.0 conditions, our results suggest that BWE is not a favorable medium for fast establishment of a biofilm.

### (ii) Quorum-sensing system expression.

In *P. aeruginosa*, biofilm formation and production of numerous virulence factors are mainly controlled by the three well-characterized quorum-sensing (QS) systems consisting of *lasI*/*lasR*, *rhlI*/*rhlR*, and *pqsH*/*mvfR* genes (27). Therefore, using quantitative reverse transcription-PCR (qRT-PCR), we decided to determine the activation of these regulatory systems by BWE (Fig. 2C). The expression levels of QS systems showed a slight reduction in LB pH 9.0 medium compared to LB pH 7.0 control conditions. Cells grown in BWE displayed, however, a slight increase in QS gene expression, mainly observed for the *Pseudomonas* quinolone system (PQS) (*pqsH*/*mvfR*). To determine whether the PQS biosynthetic pathway could be activated in BWE, we monitored the expression level of *pqsA*. Results showed a *pqsA* transcription level comparable to that seen under control conditions (Fig. 2C). The differences in the levels of QS gene expression observed between these different media were therefore not related to the alkaline pH but might suggest important characteristics of the BWE that enhance the expression of *P. aeruginosa* virulence factors. In order to further investigate the pathogenesis of *P. aeruginosa* in BWE, the major cell-associated and extracellular virulence factors were analyzed (see below).

### (iii) Pyoverdine production.

**FIG 3:**
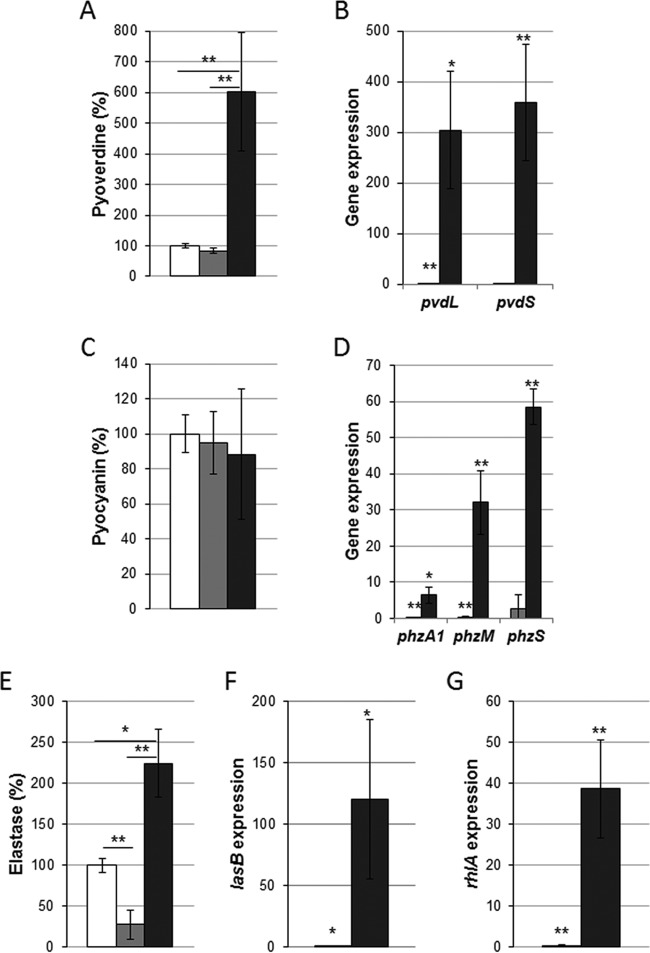
Physiology of *P. aeruginosa* PAO1 in burn wound exudate. Virulence factors pyoverdine (A), pyocyanin (C), and elastase (E) were measured in PAO1 cultures agitated for 24 h. Bacteria were grown under LB pH 7.0 conditions (white bars), LB pH 9.0 conditions (gray bars), and exudate mix conditions (black bars). Values were normalized to OD_600_, and statistics were calculated from results of three experiments performed in triplicate. Expression levels of genes involved in pyoverdine biogenesis (B), in the phenazine and pyocyanin biosynthetic pathways (D), in *lasB* elastase production (F), and in rhamnolipid biosynthesis (G) were assessed by qRT-PCR on material extracted from PAO1 cultures at an OD_600_ of 2.0. Histograms depict fold changes compared to the LB pH 7.0 conditions. Statistics are indicated using *P* of <0.05 (*) and *P* of <0.01 (**).

Previous studies on the *P. aeruginosa* gene expression profile during burn wound infection revealed an upregulation of the genes involved in iron acquisition (28). To overcome iron limitations, *P. aeruginosa* secretes siderophores such as pyoverdine to facilitate the iron uptake, which is essential for development of the bacterial population (reviewed in reference 29). Quantification, using spectrophotometric assay, showed a strong induction of pyoverdine production by *P. aeruginosa* proliferating in the exudate mix (Fig. 3A). This analysis was also confirmed at the gene expression level by qRT-PCR, showing an upregulation of *pvdS* and *pvdL* pyoverdine biosynthesis genes (Fig. 3B). These data suggest that iron availability might be limiting in exudate mixes, forcing *P. aeruginosa* to overproduce siderophores to grow.

### (iv) Pyocyanin production.

The cytotoxic blue pigment pyocyanin was shown to block wound healing by promoting the establishment of oxidative stress conditions and p38 mitogen-activated protein kinase (MAPK) pathway activation in infected tissues (30). *P. aeruginosa* PAO1 pyocyanin production was measured from culture supernatants after 24 h of growth in BWE. The pigment level remained unchanged compared to that seen under both LB control conditions (Fig. 3C). Because of the high signal background in the BWE negative control, we decided to monitor by quantitative PCR (qPCR) the expression of the *phzA1*, *phzM*, and *phzS* genes, involved in pyocyanin biosynthesis (31). Results indicated an induction of the pyocyanin biosynthesis pathway in the presence of BWE (Fig. 3D).

### (v) Elastase activity.

During burn wound infections, *P. aeruginosa* further damages epithelial tissues through the secretion of proteases, such as elastase, encoded by the QS-regulated gene *lasB* (32). LasB elastase belongs to the zinc metalloproteases family and plays a central role in colonization and destruction of host tissue by degrading elastin (33). To test whether BWEs trigger elastase production by *P. aeruginosa*, a proteolytic activity measurement was performed on culture supernatants using the elastin Congo red (ECR) assay (34). Results showed a more than 2-fold increase of the elastase activity in the exudate mix compared to the LB pH 7.0 control conditions (Fig. 3E). However, this activity displayed a 3-fold reduction under the LB pH 9.0 growth conditions. It is to be noticed that the absolute elastase activity values in BWE prior *P. aeruginosa* inoculation were already 1.4 times higher than those found under the LB pH 7.0 conditions after 24 h. This might have been due to the release of host proteases at the wound site as described previously (35). Elastase activity measurements were reinforced by the results of transcriptional analysis, since a 120-fold induction of *lasB* expression in the exudate mix was observed (Fig. 3F). Taken together, these data highlight the strong production of elastin-degrading enzymes by *P. aeruginosa*, which may contribute to further damage in the burn wound.

### (vi) Rhamnolipid biosynthesis.

Rhamnolipids are involved in *P. aeruginosa* swarming motility and in the dynamics of biofilm structures (36). They have also been shown to alter epithelial integrity by targeting cell junctions, leading to an increase in pathogen dissemination (37). To evaluate whether bacteria activate the pathway leading to production of rhamnolipids while growing in BWE, the expression level of *rhlA*, involved in biosynthesis of rhamnolipids, was monitored (Fig. 3G). qRT-PCR analysis revealed a strong stimulation of *rhlA* gene expression in the exudate mix, suggesting a contribution of biosurfactants to bacterium growth in the burn wound environment.

Taken together, the results of our analysis of *P. aeruginosa* virulence factor production revealed a moderate activation of QS systems in association with strong expression of target genes in BWE.

### Physicochemical characterization of burn wound exudate.

**FIG 4:**
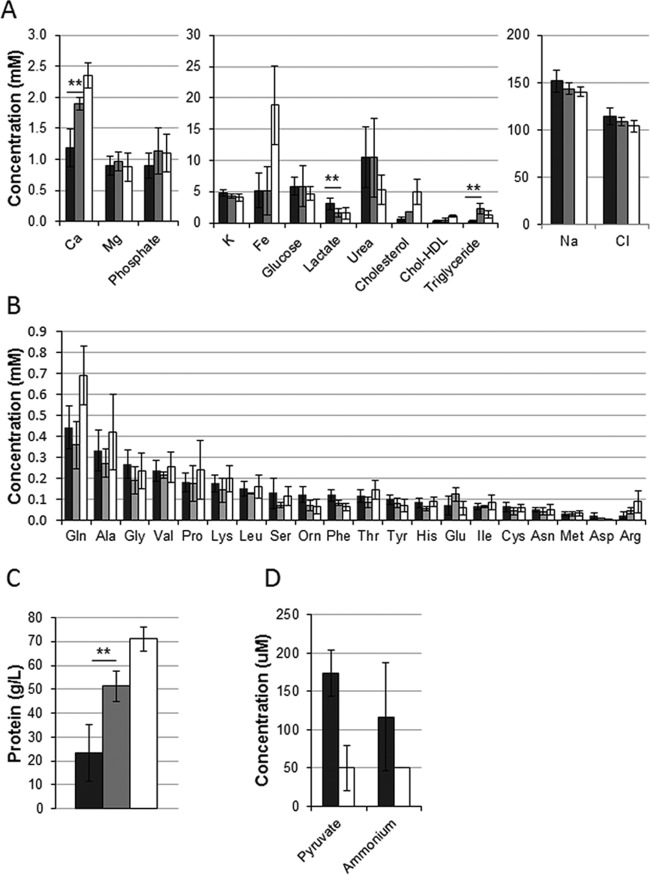
Chemical composition comparison of BWE and burn human serum and clinical reference values. (A to D) Analysis and statistic calculations were done on 15 BWE samples from 4 patients (black bars) and on 3 different burn patient sera (gray bars). The clinical reference values and their corresponding ranges are indicated by white bars. The analyses include determination of chemical composition (A and D), amino acids (B), and protein concentrations (C). Statistics are indicated using *P* of <0.01 (**). Data for pyruvate and ammonium concentrations (D) in the serum of burn patient were not available.

In order to characterize the chemical composition of the BWE, analysis of 15 exudates was performed, including the measurement of the concentrations of the main electrolytes (e.g., Fe^2+^, Mg^2+^, Cl^−^, Na^+^), lipids, proteins, and amino acids (see Table S1 in the supplemental material). Moreover, each single exudate was recorded for its visual aspect and pH value (see Fig. S1B in the supplemental material). Component concentrations obtained in BWE were compared to those in burn patient sera in order to identify specific compound depletion or enrichment results. Composition analysis data were further compared to clinical reference values in order to provide information on metabolic modifications induced by burn trauma. BWE data analysis revealed some variations in compound concentrations among the exudates (Fig. 4) that might depend on patient history (see Table S1). Nevertheless, no major differences were observed between exudates sampled from antibiotic-treated patients and those sampled from untreated patients (see Fig. S1B).

10.1128/mSphere.00111-15.5 Table S1  Chemical composition of the burn wound exudate. Download Table S1, DOCX file, 0.1 MB. Copyright © 2016 Gonzalez et al. 2016 Gonzalez et al. This content is distributed under the terms of the Creative Commons Attribution 4.0 International license.

Observation of the ion concentrations in BWE showed an iron level three times lower than the average clinical reference value (Fig. 4A). Observed over time, iron levels in exudates as well as hemoglobin levels in burn patient sera were decreasing in all the patients (see Fig. S4 in the supplemental material). Considering BWE as a potential growth medium for pathogenic bacteria, the iron level was not comparable to what was found under iron-starved conditions, despite the strong induction of pyoverdine observed (Fig. 3A and B). The constant levels of Na^+^ and Cl^−^ ions (Fig. 4A) were consistent with the saline reanimation protocol used as the standard of care. The glucose levels observed both in BWE and in burn patient sera showed similar and normal values consistent with glucose control protocols (Fig. 4A). Measurement of concentrations of cholesterol and triglycerides suggests that lipids were not lost from burn patient serum via BWE production (Fig. 4A). Glutamine was the only amino acid with a reduced concentration in the BWE and in the burn patient serum compared to clinical reference values (Fig. 4B). The total protein concentration was also strongly reduced in the exudate compared to burn patient sera (Fig. 4C). This parameter, in addition to the increase of the pyruvate concentration measured in BWE, is an important factor for the growth rate of *P. aeruginosa* (Fig. 4D)*.*

10.1128/mSphere.00111-15.4 Figure S4  Evolution of serum hemoglobin levels and exudate iron concentrations over time in burn patients. (A) Hemoglobin levels were quantified in sera collected from burn patients 3 (◆), 4 (●), 6 (▲), and 11 (■). (B) Iron concentrations were measured in samples of exudates collected from the same corresponding burn patients. Download Figure S4, TIF file, 0.1 MB. Copyright © 2016 Gonzalez et al. 2016 Gonzalez et al. This content is distributed under the terms of the Creative Commons Attribution 4.0 International license.

## DISCUSSION

Severe burns are very devastating forms of trauma which require immediate and specialized medical care. The immunosuppression state, triggered by the burn trauma, and the wound local microenvironment are favorable elements for microbial colonization and proliferation (38–40). Among the burn wound pathogens, the Gram-negative bacterium *P. aeruginosa* presents the highest incidence and becomes, generally, predominant in developed infections (11, 41). In this study, we investigated *in vitro* the physiology of the burn wound isolate and multidrug-resistant strain *P. aeruginosa* PAO1, growing in patient burn wound exudate (BWE). Additionally, we performed a chemical composition analysis of the BWE to understand its impact on *P. aeruginosa* and the clinical significance for the patient health status.

The *P. aeruginosa* PAO1 proliferation in BWE, collected from patients prior antibiotic treatment, is characterized by slow growth kinetics, whereas no bacterial growth was observed for the Gram-negative *A. baumannii* strain and *E. coli* or the Gram-positive *S. aureus* strains, suggesting an inhibitory effect of BWE. In the case of both *S. aureus* strains tested, the number of bacterial cells was even reduced after incubation in BWE, indicating some medium-mediated bacterial killing. The methicillin-resistant *S. aureus* USA300 strain was previously reported to trigger infection in mouse burn wounds (42). Nevertheless, the particularity of rodent burn models is in the absence of exudate production at the wound site (43). These results support our observation of an antibacterial activity mediated by the BWE. However, the exact trigger of this antibacterial activity is not currently identified, and the activity may have a multifactorial origin, with a combination of immune molecule activities, redox properties, and reduced ion and nutrient availability in BWE. The last parameter is critical to successful development of pathogenic bacteria, such as *P. aeruginosa*, during the infection process (44–48).

Iron represents an essential element for pathogen proliferation; however, its accessibility is limited in the host organism because of complex formation with proteins or heme molecules. Acquisition of iron is therefore considered a major challenge for host-invading microorganisms. To overcome iron limitation, *P. aeruginosa* produces various pigments with siderophore properties such as pyoverdine or pyochelin (49). These molecules are secreted by the bacterium to the outside, where they bind to iron atoms before being taken up again. We have shown here that, while growing in human burn wound exudates, *P. aeruginosa* strongly induces the production of pyoverdine (Fig. 3A and B). This is consistent with a recently reported transcriptomic study that highlighted similar gene expression profiles in *P. aeruginosa* growing in a mouse burn model and under *in vitro* iron-depleted conditions (28). Disruption of the host iron homeostasis by *P. aeruginosa* can lead to a hypoxic response, as described in the *Caenorhabditis elegans* infection model (50). In order to increase iron availability, *P. aeruginosa* also uses secreted proteases, such as elastase, that contribute to host tissue destruction, with subsequent release of iron to the pathogen proximity. Our results show a stimulation of extracellular protease production in *P. aeruginosa* growing in BWE (Fig. 3E and F). This was confirmed by gene expression analysis, where a strong induction of *lasB* could be observed. Interestingly, the LasI/LasR (LasI/R) QS system, known to control *lasB* expression, displayed only moderate induction in *P. aeruginosa* growing in human BWE (Fig. 2C). Similar results were reported in the mouse infection model, with almost no activation of *P. aeruginosa* QS systems (28). Interestingly, the expression of *rhlA* encoding the production of rhamnolipids is strongly induced in *P. aeruginosa* cultured in BWE (Fig. 3G) despite reduced stimulation of the RhlI/R QS system (Fig. 2C).

The measurement of biofilm formation, a QS-controlled phenotype, revealed that the amount of biofilm produced by *P. aeruginosa* while growing in BWE was lower than the amount seen under LB pH 7.0 control conditions. Although the bacteria conserve the capacity to form biofilm in BWE, the reduced quantity formed might be of clinical importance. These physiological observations suggest that in BWE, *P. aeruginosa* PAO1 bacteria are in an invasive state characterized by the strong production of factors involved in virulence and colonization of the host organism. This interpretation is strengthened by the finding of slower growth kinetics of PAO1 in BWE compared to control conditions. Moreover, the BWE composition may itself strongly influence the dynamic and extent of biofilm formation. Nevertheless, the gene expression analysis revealed a slightly higher level of activation of the PQS system under BWE conditions but not of the PQS biosynthesis operon (Fig. 2C). These results suggested that the regulation of PQS biosynthesis in this biological fluid is more complex than is what observed in LB medium as reported *in vitro* with cystic fibrosis (CF) sputum (44). Moreover, depending on the carbon source, *P. aeruginosa* is able to activate QS-dependent mechanisms without visible induction of major upstream systems LasI/R and RhlI/R (48).

Overall, our results highlight a stimulation of virulence factor production in *P. aeruginosa* PAO1 growing in human BWE, combined with the levels of expression of major QS systems, LasI/R, RhlI/R, and PqsH/MvfR, which remain close to control condition levels. BWE allows *P. aeruginosa* to produce all the factors required to initiate a successful infection, going from tissue destruction to acquisition of essential elements, such as iron, and reduction of surface tension via the production of biosurfactants. All these observations provide information crucial to understanding and developing new and novel strategies to prevent and treat *P. aeruginosa* infections in the severe-burn patient. Approaches targeting formation of biofilms and quorum-sensing signaling pathways may be efficiently improved by an inactivation of downstream virulence factors such as those involved in iron acquisition.

Unlike that of other extensively studied biological fluids such as CF sputum (22), the composition of BWEs has remained only poorly or partially described (19, 21). The overall chemical analysis performed in this study showed that the composition of BWE is similar to that of the burn patient serum except for a few elements such as calcium, lactate, and lipids. The increased lactate concentration (Fig. 4A) may be more a consequence of a glucose metabolism defect than a marker of hypoxia (51), while the high level of pyruvate measured in BWE (Fig. 4D) might be caused by the uncoupled oxidation occurring in mitochondria after burn trauma, as reported previously (52, 53). Taking the data together, this chemical analysis highlights the strong metabolic derangement caused by severe burn injuries. Burn trauma is often associated with transient insulin resistance (54), which leads to an increase in serum glucose concentrations and positively correlates with infections and mortality (55). Nevertheless, the glucose level observed in BWE was within the clinical reference range, which is consistent with the glucose control protocol (Fig. 4A).

The low concentration of cholesterol and triglycerides measured in BWE suggests that these lipids are not released from burn wounds (Fig. 4A). Interestingly, a low level of plasma cholesterol and an increase of levels of triglycerides are positively correlated with higher mortality in burn patients and therefore represent important parameters for the health status evaluation (56). The increase in lipoprotein catabolism and the release of free fatty acids by lipid storage lipolysis are, respectively, responsible for a decrease in cholesterol concentrations and an increase of levels of triglycerides in burn patient plasma (56).

The low level of glutamine in BWE is of particular interest since it explains the general depletion of this amino acid in burn wound patients (57). Low levels of glutamine are known to impair immunity mediated by lymphocytes and macrophages (reviewed in reference 58). The high rate of increase in protein breakdown after burn trauma observed in people recovering from burn injuries (59, 60) may explain the high level of urea (Fig. 4A) measured in both burn patient serum and BWE, which could participate in the general loss of nitrogen in burn patients (60). Taking the results together, the analysis of BWE and burn patient serum revealed strong similarities in their chemical compositions. Moreover, the data confirm the hypermetabolic state of severely burned patients and provide an explanation for the low level of essential elements such as glutamine and iron.

The chemical composition of BWE and the physiology of *P. aeruginosa* while growing in it are the basis for the formulation of an artificial burn wound exudate medium (ABWEM). This medium will be of high interest in efforts to establish an *ex vivo* burn wound infection model and to avoid the problems due to limited access to patient exudates. The ABWEM would be an efficient tool to investigate the antibacterial activity present in BWE.

## MATERIALS AND METHODS

### Bacterial strains and growth assay.

*P. aeruginosa* strains PAO1 and PA14 and clinical isolate *P. aeruginosa* PA25688, *E. coli* MG1655, *S. aureus* ATCC 29213, *S. aureus* USA300, and *A. baumannii* ATCC 19606 were cultured at 37°C in Luria-Bertani (LB) medium (United States Biological) or in human burn wound exudates (BWEs) (for collection details, see below). Due to the basic pH value of the exudate mix, the LB medium was adjusted to pH 7.0 and pH 9.0 for control conditions.

For growth experiments, overnight cultures were diluted to an optical density at 600 nm (OD_600_) of 0.05 in 96-well plates with 200 µl medium per well. Bacteria were incubated at 37°C with agitation unless mentioned otherwise. Cultures (OD_600_) were monitored over time using a Microplate reader (Biotek Instruments) or a shaking incubator (Shel Lab).

### Human burn wound exudate collection and chemical analysis.

Burn wound exudates were collected at the Burn Care Unit of the Lausanne University Hospital from February to October 2014 from 5 consecutive patients admitted for burn trauma. The study was accepted by the State Ethics Commission for human research (protocol 488/13), and the collected BWE samples were regulated by Biobank B5 of the Burn Center under the same accepted protocol. The Institutional Review Board (Commission cantonale d’Ethique du Canton de Vaud) approved the study and waived the need for informed consent.

Exudates were collected from the day of patient admission until natural arrest of exudation or surgical closure of the wound bed by skin grafting, whichever came first (submitted for publication). In brief, the wound was disinfected with 0.05% chlorhexidine solution and was abundantly rinsed with sterile 0.9% NaCl. The wound bed was then partially covered with a silicon film folded in two, onto which a silicon drain was placed. The exudate collection area was sealed with an occlusive plastic dressing. The drain was connected to a sterile plastic bottle (reservoir) and to mural suction. A continuous negative pressure was applied at 125 mm Hg. The exudate aspirated into the reservoir was collected twice a day (morning and evening) by changing the bottle. The dressing was changed at each patient shower (every 48 to 72 h). Burn wound exudates used in this study were selected from patients based on the following criteria: (i) no initial bacterial infection, (ii) no HIV, and (iii) no HBV infections. A total of 15 collected samples were stored at −80°C until further chemical analysis. The seven BWE samples, collected from patients under neither antibiotic prophylaxis nor antibiotic treatment, were mixed in equal volumes to produce the BWE mix used for bacterial growth and physiology study.

### Physicochemical analysis of biological fluids and measurement of trace elements.

Chemical analysis of the BWE and burn patient sera was performed at the Laboratory of Clinical Chemistry at Lausanne University Hospital (CHUV) on 15 BWEs (see Fig. S1 in the supplemental material). Data were compared to the clinical reference values. Trace elements of the BWE were analyzed by inductively coupled plasma mass spectrometry (ICP-MS) at the University Centre of Legal Medicine, Lausanne-Geneva. The pH was measured using an ultraBasic benchtop pH meter coupled to an InLab Micro electrode (Mettler-Toledo).

### Biofilm quantification.

Bacteria were cultured in 96-well plates containing 200 µl medium per well under static conditions for 24 h at 37°C. For quantification, planktonic cells were plated on solid medium after serial dilution and incubated overnight at 37°C prior counting. Quantification of adherent bacteria was performed as described previously (61). Briefly, adherent cells were washed twice with H_2_O–0.9% NaCl before treatment with 5 mg/ml cellulase for 1 h at room temperature under shaking conditions. Cells were then serially diluted, plated, and incubated, followed by counting the day after.

Biofilm were quantified according to a previously described protocol (62) with some adaptations. Planktonic cells were removed, and sessile cells, forming the biofilm, were fixed with methanol (99%) for 30 min prior washing with distilled water. Biofilm were stained with an aqueous solution of 1% crystal violet for 30 min. The excess crystal violet was discarded, and wells were rinsed with water. Stained biofilms were resuspended in 33% acetic acid, and absorbance was measured at 590 nm by spectrophotometry. Assays were performed in triplicate in three independent experiments, and standard deviations (and error bars) are indicated.

### Pyoverdine measurement.

Bacteria were cultured in a 96-well plate with agitation for 24 h at 37°C. Cells were removed by centrifugation, and 100 µl culture supernatant was analyzed using a spectrofluorimeter with excitation at a wavelength of 398 nm and emission at a wavelength of 447 nm (63). The LB pH 7.0 condition was used as a reference. Experiments were performed three times in triplicate, and standard deviations (and error bars) are indicated.

### Pyocyanin concentration measurement.

Pyocyanin production was evaluated as previously described (64). Briefly, bacteria were incubated in a 96-well plate with agitation for 24 h at 37°C. Cells were removed by centrifugation, and absorbance at 691 nm was measured in 100-µl culture supernatant after vortex mixing. Data were normalized to the background, and the LB pH 7.0 value was used as a reference. Analyses were performed in triplicate in three independent experiments, and standard deviations (and error bars) are indicated.

### Elastase activity assay.

Measurement of elastase activity was performed according to the elastin Congo red (ECR) assay previously described in reference 34. Briefly, 10 µl of culture supernatant was mixed with 190 µl of ECR buffer composed of Tris-HCl (100 mM), CaCl_2_ (1 mM, pH 7.5), and 20 mg/ml of elastin Congo red (Sigma). After agitation 1 h at 37°C, the insoluble ECR was removed by centrifugation (5 min, 2,500 × *g*). Absorbance of the supernatant was measured at 495 nm by spectrophotometry. Data were normalized to the control background prior normalization to the OD values. Analyses were performed three times in three biological replicates, and standard deviations (and error bars) are indicated.

### Gene expression analysis.

RNA extraction was performed on cultures at an OD_600_ of 2.0 grown in 200 µl exudate mix. Each replicate was constituted by a pool of 5× 200 µl culture and treated with RNA Protect bacterial solution (Qiagen) prior centrifugation and storage at −20°C. Then, cell pellets were lysed with lysozyme (1 mg/ml) in Tris-EDTA (TE) and total RNA was extracted using an RNeasy column (Qiagen) according to the manufacturer’s instructions. Purified RNA was eluted in 50 µl RNase-free water, and the concentration was quantified using a Qubit fluorometer (Life Technologies). DNase treatment was carried out with RQ1 RNase-free DNase according to the instructions furnished by the manufacturer (Promega). For cDNA synthesis, 500 ng of DNase-treated total RNA was reverse transcribed using random hexamer primers (Promega) and Improm-II reverse transcriptase (Promega) according to the protocol instructions. Reverse transcriptase was heat inactivated prior qPCRs performed on cDNA using SYBR green (Thermo Scientific). Primers used for the RT-PCR analyses are listed in Table S2 in the supplemental material. Data analysis was performed according to the method described in reference 65, and the *oprF* gene was used as an internal control. Analysis was done in duplicate in three independent experiments, and standard deviations (and error bars) are indicated.

10.1128/mSphere.00111-15.6 Table S2  Primers used in this study. Download Table S2, DOCX file, 0.01 MB. Copyright © 2016 Gonzalez et al. 2016 Gonzalez et al. This content is distributed under the terms of the Creative Commons Attribution 4.0 International license.
