# Supplementary figures and images for: Effect of Human Burn Wound Exudate on Pseudomonas aeruginosa Virulence
Source: mSphere. 2016 Apr 27;1(2):e00111-15. doi: 10.1128/mSphere.00111-15 (PMC4894682; doi:10.1128/mSphere.00111-15)

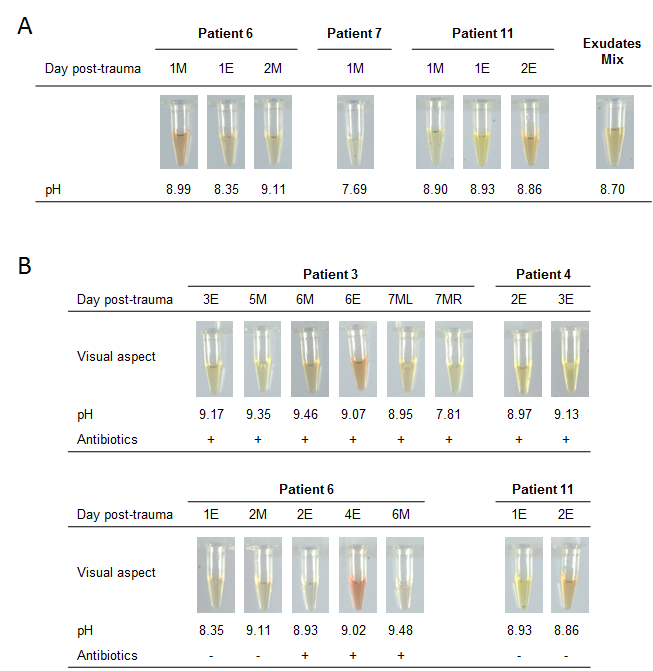

Supplement: Figure S1 [file sph002162073sf3.tif]

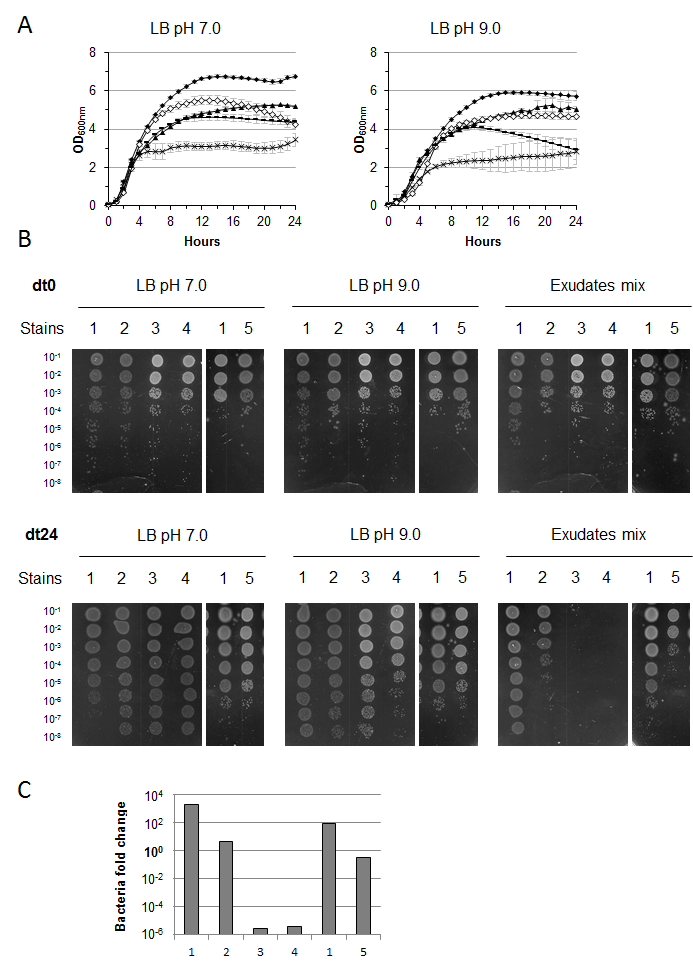

Supplement: Figure S2 [file sph002162073sf4.tif]

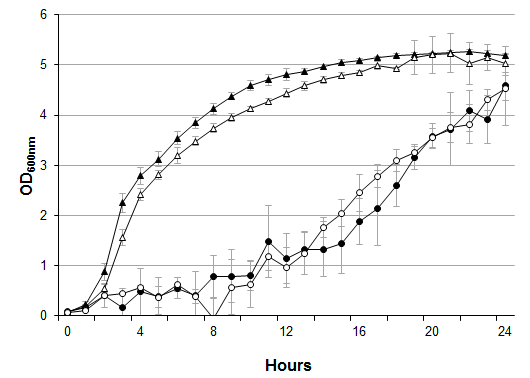

Supplement: Figure S3 [file sph002162073sf5.tif]

**Table S1. Chemical composition of the burn wound exudate.**


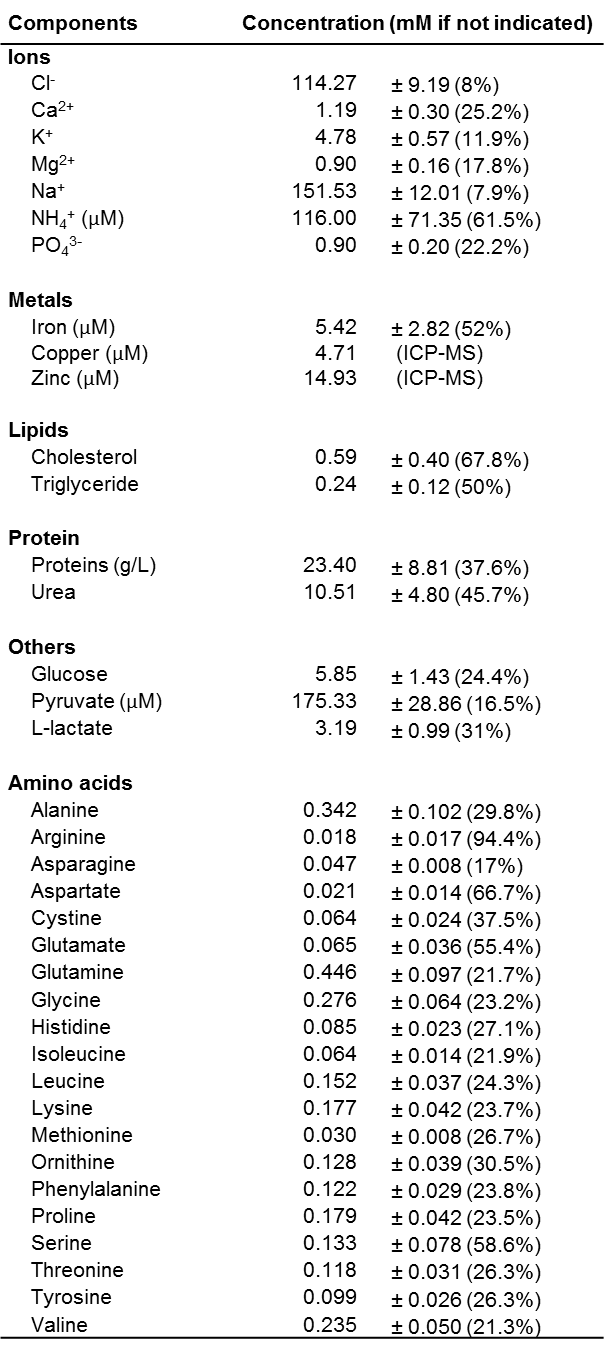

Supplement: Table S1 [file sph002162073st1.docx]

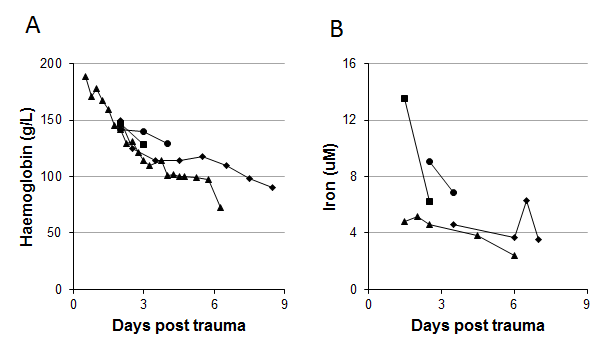

Supplement: Figure S4 [file sph002162073sf6.tif]
