# Supplementary material for: Effect of Human Burn Wound Exudate on Pseudomonas aeruginosa Virulence
Source: mSphere. 2016 Apr 27;1(2):e00111-15. doi: 10.1128/mSphere.00111-15 (PMC4894682; doi:10.1128/mSphere.00111-15)
Supplement: Table S2 [file sph002162073st2.docx]

**Table S2. Primers used in this study.**

| **Gene** | **PA number** | **Primers sequences (5’🡪3’)** |
| --- | --- | --- |
| *lasI* | PA1432 | CTACAGCCTGCAGAACGACA |
|  |  | ATCTGGGTCTTGGCATTGAG |
| *lasR* | PA1430 | ACGCTCAAGTGGAAAATTGG |
|  |  | GTAGATGGACGGTTCCCAGA |
| *rhlI* | PA3476 | CTCTCTGAATCGCTGGAAGG |
|  |  | GACGTCCTTGAGCAGGTAGG |
| *rhlR* | PA3477 | AGGAATGACGGAGGCTTTTT |
|  |  | CCCGTAGTTCTGCATCTGGT |
| *pqsA* | PA0996 | CAATACACCTCGGGTTCCAC |
|  |  | TGAACCAGGGAAAGAACAGG |
| *pqsH* | PA2587 | ATGTCTACGCGACCCTGAAG |
|  |  | AACTCCTCGAGGTCGTTGTG |
| *mvfR* | PA1003 | AACCTGGAAATCGACCTGTG |
|  |  | TGAAATCGTCGAGCAGTACG |
| *pvdL* | PA2424 | ACCCTGCGTGCTGATGTC |
|  |  | TCGGCTCGGAACCGGAGAA |
| *pvdS* | PA2426 | AGATCACTTCGTCGTTCAAGGCA |
|  |  | GATGTGTTCGAGGGTCGCGTA |
| *phzA1* | PA4210 | AACGGTCAGCGGTACAGGGA |
|  |  | GTGGGAATACCGTCACGTTT |
| *phzM* | PA4209 | CTGCTGCGCGTAATTTGATA |
|  |  | AGATCTCGAAGGCCACCAG |
| *phzS* | PA4217 | CGTCGGCATCAATATCCAG |
|  |  | AGTACTGCGGATAGGCGTTG |
| *lasB* | PA3724 | AAGCCATCACCGAAGTCAAG |
|  |  | GTAGACCAGTTGGGCGATGT |
| *rhlA* | PA3479 | CGAGGTCAATCACCTGGTCT |
|  |  | GACGGTCTCGTTGAGCAGAT |
| *oprF* | PA1777 | GGTTACTTCCTGACCGACGA |
|  |  | TCGCTGTTGATGTTGGTGAT |
